# Lab-based semen parameters as predictors of long-term health in men—a systematic review

**DOI:** 10.1093/hropen/hoae066

**Published:** 2024-11-08

**Authors:** Silvia Nedelcu, Srisailesh Vitthala, Abha Maheshwari

**Affiliations:** Aberdeen Reproductive Medicine Unit, NHS Grampian, Aberdeen, UK; Institute of Applied Health Sciences, University of Aberdeen, Aberdeen, UK; MMC IVF, Dubai, United Arab Emirates; Aberdeen Reproductive Medicine Unit, NHS Grampian, Aberdeen, UK; Institute of Applied Health Sciences, University of Aberdeen, Aberdeen, UK

**Keywords:** male infertility, sperm parameters, semen analysis, cancer, cardiovascular, diabetes, mortality

## Abstract

**STUDY QUESTION:**

Can semen parameters predict long-term health outcomes in men?

**SUMMARY ANSWER:**

There is a lack of evidence to suggest a higher risk of comorbidities in men with poor semen concentration.

**WHAT IS KNOWN ALREADY:**

Male infertility has been long associated with a higher mortality risk and possibly higher chance of developing comorbidities but there has been less focus on semen analysis as a potential predictive factor.

**STUDY DESIGN, SIZE, DURATION:**

We searched PubMed/MEDLINE, EMBASE, and EBM databases from inception to December 2023. MESH term strategy: heading 1 (‘OR’, semen analysis, sperm count, sperm parameter*, male infertility, azoospermia, aspermia, oligospermia, teratozoospermia, asthenozoospermia) ‘AND’ heading 2 (‘OR’, morbidity, mortality, diabetes, cancer, cardiovascular, death, hypertension, stroke, long-term health). We included all studies that analyzed the risk of mortality and/or future development of comorbidities in men with at least one semen analysis. Case series and reviews were excluded.

**PARTICIPANTS/MATERIALS, SETTING, METHODS:**

A narrative synthesis was done for all studies and meta-analysis where possible. Odds ratio (ORs) (95% CI, *P*-value) were calculated for all men with one suboptimal semen parameter and associated with the risk of a particular outcome. The risk of bias was assessed with QUADAS-2.

**MAIN RESULTS AND THE ROLE OF CHANCE:**

Twenty-one studies were finally included. There was either a high or unclear risk of bias in all studies. The results only allowed for meta-analysis on categories of sperm concentration. We found a 2-fold increase in mortality risk in azoospermic men compared to oligospermic (OR 1.96, 95% CI: 1.29–2.96) and normozoospermic (OR 2.00, 95% CI: 1.23–3.25) groups, but not in oligospermic compared to normozoospermic (OR 1.04, 95% CI: 0.52–2.09). There was no difference in risk of cardiovascular disease in any of the sperm concentration groups (azoospermic-oligospermic OR 0.94, 95% CI: 0.74–1.20, azoospermic-normozoospermic OR 1.11, 95% CI: 0.71–1.75, and oligospermic-normozoospermic OR 1.12, 95% CI: 0.80–1.55). OR for diabetes in azoospermic men was higher only compared to oligospermic (OR 2.16, 95% CI: 1.55–3.01). The risk of all-site cancer was higher in azoospermic men compared to oligospermic (OR 2.16, 95% CI: 1.55–3.01) and normozoospermic (OR 2.18, 95% CI: 1.20–3.96). Only azoospermic men might be at higher risk of testicular cancer when compared to men with normal sperm concentration (OR 1.80, 95% CI: 1.12–2.89).

**LIMITATIONS, REASONS FOR CAUTION:**

Although our pooled analysis shows an increased risk of mortality and all-site cancer risk in azoospermic men, the results show a lack of evidence to suggest a higher risk of comorbidities in men with poor semen concentration. Given the limited available data, the nature of the studies, and the high risk of bias, the results should be interpreted with caution.

**WIDER IMPLICATIONS OF THE FINDINGS:**

There is not enough data to confirm the usability of semen analysis as a predictor of poor long-term health in men, especially within the general population.

**STUDY FUNDING/COMPETING INTEREST(S):**

No funding was obtained for this study. A.M. has received funding from Merck Serono, Ferring, Gedeon Richter, Pharmasure, and Cook Medical to attend medical conferences; has been a participant in an advisory board for Ferring; and has given an invited lecture for a Merck Serono advisory board. S.N. has received funding for medical conference attendance from Ferring and Cook Medical.

**REGISTRATION NUMBER:**

PROSPERO No. CRD42024507563.

WHAT DOES THIS MEAN FOR PATIENTS? Infertility is defined as the failure to achieve a pregnancy after at least 1 year of regular unprotected sexual intercourse. One in two infertile couples will have a male cause. Male infertility can occur through failure of sperm production, delivery, or both, and one way of assessing sperm production is by taking a sperm sample.

There is now emerging evidence that sperm tests can work as a predictor of long-term health conditions, through mechanisms that remain largely unknown. We systematically looked at the literature and identified 21 studies. After combining the data, we observed a higher chance of death and cancer in men who do not produce any sperm (azoospermic).

Couples diagnosed with infertility are not routinely followed up after being offered fertility treatment. However, given that azoospermic men might have a higher risk of poor long-term health, they might benefit from counseling and routine screening in a primary care setting.

Further good-quality studies are needed before one can advise on the implications of a suboptimal semen result on the long-term health of men with any degree of confidence.

## Introduction

Infertile couples are investigated and treated in a clinical setting and usually their care ends there. However, the American Society of Reproductive Medicine (ASRM) states that male assessment has the scope of identifying any conditions that can affect the health of both patient and their offspring (Schlegel *et al.*, 2021). The potential effect of reproduction capacity on mortality has been studied since the 1990s (Friedlander, 1996), but not until the early 2000s, has the possible impact of infertility and abnormal sperm analysis come to the public’s attention (Groos *et al.*, 2006). More recently, there has been new data accrual suggesting that infertile men are at a higher risk of developing chronic diseases, especially cardiovascular diseases (CVDs), cancers, and diabetes (del Giudice *et al.*, 2020).

Globally, the prevalence of male infertility has increased by 76.9% in three decades, with the highest among men aged 30–34 years (Huang *et al.*, 2023). This implies that more men in their 30s are being assessed for infertility, when, age-wise, the risk of having associated comorbidities is lower. In a prospective study of 1737 patients with reduced sperm counts (Punab *et al.*, 2017), 60% of infertile, 17.3% of azoospermic, and 75% of oligospermic men did not have an identifiable medical causal factor for their infertility. Based on some of the most recent studies, infertile men are at higher risk of mortality and poor health in the future. However, in a clinical setting, the question remains, as to what extent we can advise patients on their risk of mortality or poor long-term health, based on semen analysis or their diagnosis of male infertility.

There is a growing body of evidence supporting the theory that male infertility mirrors the co-existence and future development of adverse health outcomes (Choy and Eisenberg, 2018), independent of putative ‘contributing’ factors to low semen quality, such as obesity or pre-existing medical conditions. This can be observed in the multitude of reviews that have looked into the association between clinical male infertility, involuntary childlessness, and long-term health outcomes in men (Mao *et al.*, 2016; Glazer *et al.*, 2017a; del Giudice *et al.*, 2020, 2021a,b; Behboudi-Gandevani *et al.*, 2021; Laukhtina *et al.*, 2021; Fallara *et al.*, 2024). However, there are several shortcomings of the existing literature on the matter: (i) there is significant variation in how male infertility has been defined over time (Agarwal *et al.*, 2015); (ii) there is considerable selection bias as most subjects are being investigated for couple infertility in a clinical setting, which is not representative of the general population; (iii) studies including men with involuntary childlessness are less reliable as they do not confidently exclude potential female factors; and (iv) retrospective data collection increases bias and limits statistical interpretation.

Semen analysis is an objective method to assess male infertility. There are several studies that have focused on semen analysis as a marker of male infertility and the potential association with adverse long-term health outcomes. Therefore, we have performed a systematic review to answer the question whether semen analysis could be used as a predictive test for specific comorbidities.

## Methods

### Data collection and inclusion criteria

The literature search included PubMed/MEDLINE, EMBASE, and EBM (including Cochrane library) as main databases, starting from their inception to December 2023. There was no language restriction. The reference lists of all included studies were manually searched, as were in press articles from major Reproductive Medicine and Andrology journals. All relevant reviews and meta-analyses were extracted separately and their references were manually searched for any titles that might have been missed from the main database search.

Any study that analyzed the risk of developing comorbidities or mortality risk in adult men under 50 years old with poor semen analysis was selected and extracted into Rayyan (http://rayyan.qcri.org) for full-text analysis. The age cut-off of 50 years was selected based on the notion that sperm quality significantly declines after this age and the age-related risk of comorbidities and mortality increases. Grey literature (reports, conference abstracts, theses, ongoing or abandoned clinical trials, government publications, newspaper articles, and unpublished works) was also considered for inclusion with the aim of reducing publication bias. Case series, review articles, and meta-analyses or studies that did not have semen analysis reported were excluded. The health outcome of interest had to be diagnosed after the semen analysis and during an appropriate follow-up time. The search was performed independently by two reviewers (S.N. and S.V.) and any discrepancies were resolved after discussing with A.M. The study was registered with PROSPERO: No. CRD42024507563.

### Assessment of quality of studies and risk of bias

We followed the PRISMA 2020 guideline (Page *et al.*, 2021) for this review. Each study selected for the final analysis was evaluated independently and relevant information for each study is detailed in Table 1. For the assessment of the risk of bias, we used the QUADAS-2 tool and we constructed three questions for each of the four domains (Supplementary File S1): *patient selection, index test (semen analysis), reference (outcome)*, as well as *flow and timing.* Each study was identified as having low, unclear, or high risk of bias across each domain for both bias and applicability. High risk of bias for any one domain would upgrade the overall risk. The assessment was performed independently by the two reviewers.

**Table 1.** Main findings of studies included in the analysis, separated by outcome (GCNIS, PSA, mortality, cancer, diabetes, cardiovascular disease, hospitalizations, Charlson Comorbidity Index).

**Study,** **location, timeframe**	Study design	**Population** **(number of participants)**	Main findings	Risk of bias (QUADAS2)
**Mortality**				
Groos *et al.* (2006) Germany, 1949–1985	Cross-sectional	Men who attended andrology service (1283)	No difference in mortality risk between azo-, oligo- (<20 million/ml), and normozoospermic.	High
Jensen *et al.* (2009) Denmark, 1963–2001	Retrospective cohort	Men from couples investigated for infertility (43 277); azoospermic men excluded	Mortality among infertile men, fertile men, and childless men decreased with increasing sperm concentration up to 40 million/ml (*P* trend 0.57) and with increasing motility and morphology increased (*P* trend <0.05).	Unclear
		compared to general Danish population	No difference in mortality with changes in semen volume	
Eisenberg *et al.* (2014) USA, 1994–2011	Retrospective cohort	Men from couples investigated for infertility in two cohorts, Texas and California (11 935)	SMR 0.70 (95% CI: 0.39–1.15) for concentration <15 million/ml (vs ≥15 million/ml)	Unclear
			SMR 0.55 (95% CI: 0.39–0.76) for motility <40% (vs ≥40%)	
		compared to general US population		
			SMR 0.62 (95% CI: 0.40–0.91) for TMS <9 million (vs ≥9 million)	
			SMR 0.37 (95% CI: 0.23–0.56) for morphology 14% (vs ≥14%)	
			adjusted HR if more than two abnormal parameters present 2.29 (95% CI: 1.12–4.65, *P* = 0.02)	
Glazer *et al.* (2019) Denmark, 1994–2015	Retrospective cohort	Men from couples undergoing MAR (64 563)	Azoospermic men had a higher risk of death compared to both normozoospermic (aHR 2.40, 95% CI: 1.57–3.67) and fertile men (aHR 3.66, 95% CI: 2.18–6.16)	High
		compared to age-matched fertile men (322 108)	No elevated risk of mortality among oligospermic men.	
Keihani *et al.* (2020) USA, 1996–2017	Retrospective cohort	Men from couples investigated for infertility (21 098)	Azoospermia was not associated with higher mortality or cancer risk.	High
		compared to fertile men (3026)	aHR 1.15 (95% CI: 0.75–1.77), *P*-value = 0.52 azoospermia vs fertile	
			aHR 0.89 (95% CI: 0.47–1.70), *P*-value = 0.73 azoospermia vs normozoospermic	
			aHR 1.12 (95% CI: 0.73–1.72) *P*-value = 0.59 oligospermia vs normozoospermic	
del Giudice *et al.* (2020) USA, 2003–2017	Retrospective cohort	Men diagnosed with infertility based on semen analysis (134 796)	Azoospermic men had a higher risk of all-cause mortality (aHR 2.01, 95% CI: 1.60–2.53) and cancer-related mortality (aHR 2.06, 95% CI: 1.18–3.57), compared to control.	High
		compared to men without a diagnosis of infertility (242 282)	Oligospermic men did not show a difference in mortality risk	
**Cancer**				
Jacobsen *et al.* (2000) Denmark, 1943–1995	Retrospective cohort	Men from couples investigated for infertility (32 422)	Men with concentration ≤20 million/ml (SIR 2.3, 95% CI: 1.6–3.2***), one abnormal semen parameter (SIR 1.9, 95% CI: 1.2–2.8***), two abnormal semen parameters (SIR 2.7, 95% CI: 1.1–5.5)*** and normal morphology ≤75% (SIR 1.3, 95% CI: 1.0–1.7***) had a higher risk of testicular cancer.	Unclear
		compared to general Danish population		
Eisenberg *et al.* (2013) USA, 1995–2009	Retrospective cohort	Men from couples investigated for infertility (2238) compared with general US population	Azoospermic men had a higher risk of all-site cancer compared to the general population **(**SIR 3.7, 95% CI: 1.7–7.0 and age-adjusted SIR 2.9, 95% CI: 1.4–5.4) and to non-azoospermic (aHR 2.2, 95% CI: 1.0–4.8).***	High
Hanson *et al.* (2016) USA, 1994–2011	Retrospective cohort	Men from couples investigated for infertility (20 433)	Oligospermic men (<15 million/ml) had a higher risk of all-site cancer (HR 1.7, 95% CI: 1.1–2.4) and testicular cancer (HR 11.9, 95% CI: 4.9–28.8) compared to fertile men;	Unclear
			Motility 0–49% was associated with higher risk of all-site (HR 1.5, 95% CI: 1.13–1.99) and testicular cancer (HR 1.5, 95% CI: 1.13–1.99) compared to fertile men.	
			Vitality 0–45% was associated with higher risk of all-site (HR 1.4, 95% CI: 1.0–1.9) and testicular cancer (HR 1.4, 95% CI: 1.02–1.92) compared to fertile men.	
			No difference in cancer risk for azoospermic men (HR 1.0, 95% CI 0.5–2.1).	
Keihani *et al.* (2020) USA, 1996–2017	Retrospective cohort	Men from couples investigated for infertility (21 098) compared to fertile men (3026)	No difference in cancer risk between azo-, oligo- (<20 million/ml), and normozoospermic.	High
**Diabetes**				
Eisenberg *et al.* (2016) USA, 2001–2009	Retrospective cohort	Men from couples investigated for infertility (13 027) compared to without infertility diagnosis (23 860) and vasectomized (79 099)	Men with oligospermia had a higher risk of diabetes compared to men without a diagnosis of infertility (HR 1.3, 95% CI: 1.05–1.68).***	Unclear
			Azoospermia was not associated with a higher risk of diabetes (HR 1.34, 95% CI: 0.96–0.98).	
Glazer *et al.* (2017b) Denmark, 1994–2012	Retrospective cohort	Men from couples investigated for infertility (18 499) compared to normal semen analysis or vasectomized (21 017)	Higher risk of diabetes in men with azoospermia (aHR 2.10, 95% CI: 1.25–3.56) and oligospermia (aHR 1.44, 95% CI: 1.01–2.06) compared to men with normal semen analysis or sterilized.	High
**Cardiovascular disease**				
Chen *et al.* (2022) Taiwan, 2000–2015	Retrospective cohort	Men from couples investigated for infertility (2326)	Higher risk of cardiovascular disease in men with azoospermia (aHR 1.388, 95% CI: 1.221–1.584)* and oligospermia (aHR 1.365, 95% CI: 1.191–1.562)* compared to fertile men.	Unclear
		compared to fertile men (9304)		
Eisenberg *et al.* (2016) USA, 2001–2009	Retrospective cohort	Men from couples investigated for infertility (13 027) compared to without infertility diagnosis (23 860) and vasectomized (79 099)	Azoospermia and oligospermia were not associated with a higher risk of hypertension, peripheral vascular disorder, cerebrovascular disease, or ischemic heart disease. Azoospermia was associated with a higher rate of renal disease (HR 2.26, 95% CI: 1.20–4.27).***	Unclear
**Germ cell neoplasia *in situ* (GCNIS) or Testicular carcinoma *in situ* (TCIS)**				
Pryor *et al.* (1983) UK, 1955–1982	Cross-sectional	Men from couples investigated for infertility having testicular biopsy for azoospermia or oligospermia <10 million/ml or incidental during varicocele ligation (2043)	Eight men out of 2043 were diagnosed with TCIS on biopsy and seven out of eight had sperm concentration 0–5 million/ml.	Unclear
Giwercman *et al.* (1997) Denmark, 1986–1993	Cross-sectional	Men with sperm concentration <10 million/ml in two samples within the previous 2 years or <20 million/ml in two samples within the previous 2 years with a history of cryptorchidism or one or two atrophic testicles (orchidometer volume <15 cm^3^), or both (207)	All 207 men underwent bilateral or unilateral (if one gonad present in scrotum) and no participant was positive for TCIS	Unclear
Petersen *et al.* (1999) Denmark, 1986–1993	Cross-sectional	Men undergoing contralateral orchidectomy for testicular cancer (56)	Men TCIS-positive in the contralateral testicle (25) had a lower median sperm concentration compared to TCIS-negative (31): median sperm concentration: 0.03 million/ml vs 9.1 million/ml* and lower median TSC (0.10 million vs 32 million)*	Unclear
Olesen *et al.* (2007) Denmark, 1995–2005	Cross-sectional	Men investigated for couple infertility and having testicular biopsy (453)	Ten patients were TCIS-positive on biopsy with a median sperm concentration of 0.1 million/ml (0–2.06 million/ml) and 443 patients were TCIS-negative with a median concentration of 0.16 million/ml (0–152 million/ml).	Unclear
**Prostate-specific antigen**				
Ausmees *et al.* (2014) Estonia, 2007–2010	Cross-sectional	Men undergoing prostate cancer screening (411)	Median [IQR] sperm volume, concentration, and TSC were the lowest in men with PSA ≥2.5 ng/ml compared to PSA <1 and 1.0–2.49 ng/ml.	Unclear
			median(IQR) volume(ml): 3.5(2.4–4.6),** 3.3(2.2–4.7), and 2.9(1.8–4.4)*	
			median(IQR) TSC(million): 324(169.5–519.3)*, 268(127–556),* and 198(112.9–409.2)*	
			median(IQR) concentration(million/ml): 111(51.8–176.3),* 96(36.5–163.5),* and 88.5(41.5–125.5)*	
Boeri *et al.* (2021) Italy, 2014–2019	Cross-sectional	Men from couples investigated for infertility (956)	Men with PSA >1 ng/ml had lower median [IQR] sperm concentration (8.8 million/ml [1.9–30] vs 20 million/ml [5.4–49])* and progressive motility (18% [6–30] vs 29 % [12–45])* compared to PSA ≤1 ng/ml.	Unclear
			There was no difference in volume (3 ml [2–4] vs 3 ml [1–4], *P* = 0.3) and morphology (2% [1–8] vs 2% [1–6], *P* = 0.4) between the two groups.	
**Hospitalizations**				
Latif *et al.* (2017) Denmark, 1977–2010	Retrospective cohort	Men from couples investigated for infertility (4712)	The risk of all-cause, diabetes-related, testicular-cancer related, and prostate cancer-related hospitalizations were higher in men with azoospermia and oligospermia below 15 million/ml when compared to men with sperm concentration ≥40 million/ml.	High
			CVD-related hospitalizations were only higher in oligospermic men.	
Latif *et al.* (2018) Denmark, 1977–2015	Retrospective cohort	Men from couples investigated for infertility (1423)	Men with sperm concentration below 15 million/ml and TSC below 39 million had a higher risk of hospitalizations compared to concentration above 15 million/ml and sperm count above 39 million.	Unclear
			Sperm concentration and TSC were associated with a risk of hospitalization in a dose–response pattern compared to concentration above 100 million/ml.	
**Charlson Comorbidity Index**				
Boeri *et al.* (2022) Italy, 2003–2010	Prospective cohort	Men from couples investigated for infertility (899)	Non-obstructive azoospermia status (*P* = 0.001) was independently associated with Charlson Comorbidity Index increase in a 10-year follow-up.	Unclear
			Men who had at least one basepoint increase in CCI had a lower median [IQR] sperm concentration 2 million/ml, [0–14]) compared to men without an increase in CCI (12 million/ml, IQR [1–29]) in a 10-year follow-up.	

* *P*-value < 0.001;

** *P*-value < 0.01;

*** *P*-value < 0.05.

PSA, prostate-specific antigen; CVD, cardiovascular disease; CCI, Charlson Comorbidity Index; N/A, not applicable (where there is no control group, the comparison was done between men with different sperm parameters within a cohort of men diagnosed with infertility); SMR, standard mortality ratio; SIR, standard incidence ratio; TCIS (formerly used in studies) has been recently replaced by GCNIS; IQR, interquartile range; OR, odds ratio; TSC, total sperm count; aHR, adjusted hazard ratio; HR, hazard ratio; MAR, medically-assisted reproduction.

### Data synthesis

Data synthesis was restricted by the available crude data from original papers. From each study, where possible, we extracted the number of subjects and plotted in 2 × 2 contingency tables for the studied outcome and its corresponding semen parameter. As the most studied semen parameter was concentration, we only reported results comparing groups of azoospermic, oligospermic, and normozoospermic/sterilized men. We pooled and calculated the total number of subjects within the same outcome and sperm concentration category. Odds ratio (ORs) with corresponding 95% CI with random effect were calculated to compare the risk between azoospermic, oligospermic, and normozoospermic men, for each possible outcome. Heterogeneity was assessed by calculating *I*^2^. An *I*^2^>50% suggested high heterogeneity among studies (Higgins *et al.*, 2003). Forest plot diagrams depict the results for each outcome category.

## Results

### Study selection

**Figure 1.**
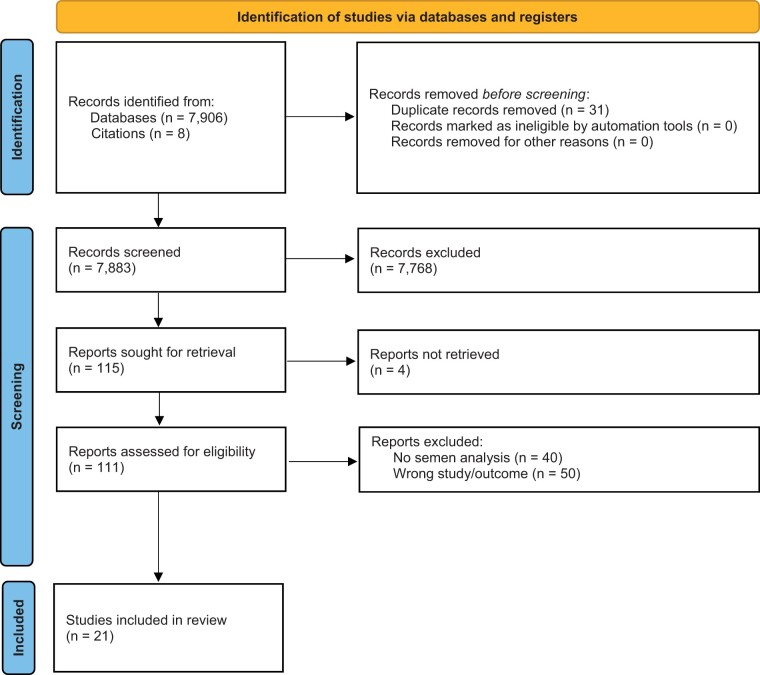
**PRISMA flowchart of the study selection process.** Twenty-one studies met all the inclusion criteria for further analysis.

The details of the study selection process are depicted in Fig. 1. The initial search was extensive and yielded a total number of 7906 studies: 4037 articles from Medline, 3401 articles from Embase, 450 from EBM reviews, and 18 from the Cochrane database. Thirty studies were either duplicates or conference abstracts of larger publications. Out of 7875 articles screened for title and abstract, 107 were sought for retrieval and full-text analysis and only 20 were included in the final review. Eight additional studies, which were manually extracted from the reference list of other papers, were sought for retrieval. Two studies were not available for full-text extraction, five did not fully meet the inclusion criteria (Skakkebæk, 1978; Nistal *et al.*, 1989; Bettocchi *et al.*, 1994; Pasqualotto *et al.*, 2003; Mancini *et al.*, 2007) and one was added to the final analysis (Pryor *et al.*, 1983). In total, 21 studies were included in the systematic review.

### Risk of bias

None of the studies were at low risk of bias (Table 1). Seven studies were classified as having a high risk of bias and 14 with unclear risk. We appreciate that this scoring resulted mainly from population selection. The methodology for the risk assessment and results are detailed in Supplementary File S1. The studies were also subject to reporting bias due to their predominantly retrospective nature, and we also identified location bias (15 studies conducted in the USA and Denmark).

### Study characteristics

Descriptions of the main characteristics of the studies are detailed in Table 1. Concentration was the predominant parameter reported. Fourteen were retrospective longitudinal cohort studies (Jacobsen *et al.*, 2000; Groos *et al.*, 2006; Jensen *et al.*, 2009; Eisenberg *et al.*, 2013, 2014, 2016; Hanson *et al.*, 2016; Glazer *et al.*, 2017b, 2019; Latif *et al.*, 2017, 2018; del Giudice *et al.*, 2020; Keihani *et al.*, 2020; Chen *et al.*, 2022), one was a prospective longitudinal cohort study (Boeri *et al.*, 2022) and six were cross-sectional studies (Pryor *et al.*, 1983; Giwercman *et al.*, 1997; Petersen *et al.*, 1999; Olesen *et al.*, 2007; Ausmees *et al.*, 2014; Boeri *et al.*, 2021). One study was an abstract (Keihani *et al.*, 2020) with no publication following. Both articles published by Glazer *et al.* (2017b, 2019) report their studies being prospective cohort studies, but data were historical and extracted from registries predating the study.

Study settings spanned across three continents: six studies were conducted in the USA (Eisenberg *et al.*, 2013, 2014, 2016; Hanson *et al.*, 2016; del Giudice *et al.*, 2020; Keihani *et al.*, 2020). Out of 14 studies taking place in Europe, nine were in Denmark (Giwercman *et al.*, 1997; Petersen *et al.*, 1999; Jacobsen *et al.*, 2000; Olesen *et al.*, 2007; Jensen *et al.*, 2009; Glazer *et al.*, 2017b, 2019; Latif *et al.*, 2017, 2018), two in Italy (Boeri *et al.*, 2021, 2022), one in Germany (Groos *et al.*, 2006), one in the UK (Pryor *et al.*, 1983), one in Estonia (Ausmees *et al.*, 2014) and only one study in Asia and Taiwan each (Chen *et al.*, 2022). Three studies were population-based, using national health insurance databases to select men diagnosed with infertility; 17 were clinic-based (andrology or infertility clinics) and 1 was hospital based. Keihani *et al.* (2020) do not mention the data collection source, however, infertile patients were further linked to the Utah Population Database for outcome search.

A total of 12 studies were from a single center (Pryor *et al.*, 1983; Jacobsen *et al.*, 2000; Groos *et al.*, 2006; Olesen *et al.*, 2007; Jensen *et al.*, 2009; Eisenberg *et al.*, 2013; Ausmees *et al.*, 2014; Hanson *et al.*, 2016; Latif *et al.*, 2017, 2018; Boeri *et al.*, 2021, 2022). From the non-population-based studies, four were multicenter (Giwercman *et al.*, 1997; Eisenberg *et al.*, 2014; Glazer *et al.*, 2017b, 2019) and two did not provide further details on the setting (Petersen *et al.*, 1999; Keihani *et al.*, 2020). Ausmees *et al.* (2014) investigated a cohort of men who attended the Andrology Centre for prostate health screening. All other studies included men initially assessed for couple infertility. Sample sizes varied from cohorts of 54 (Petersen *et al.*, 1999) to 384 419 subjects (Glazer *et al.*, 2019). The studies analyzed one or multiple outcomes: all-cause mortality (Jensen *et al.*, 2009; Eisenberg *et al.*, 2014; Glazer *et al.*, 2019; del Giudice *et al.*, 2020; Keihani *et al.*, 2020) or lifespan (Groos *et al.*, 2006), hospitalizations (Latif *et al.*, 2017, 2018), cancer (Jacobsen *et al.*, 2000; Eisenberg *et al.*, 2013; Hanson *et al.*, 2016; Latif *et al.*, 2017; Keihani *et al.*, 2020), CVD (Eisenberg *et al.*, 2016; Latif *et al.*, 2017; Chen *et al.*, 2022), diabetes (Eisenberg *et al.*, 2016; Glazer *et al.*, 2017b; Latif *et al.*, 2017), Charlson Comorbidity Index (CCI) (Boeri *et al.*, 2022), germ cell neoplasia *in situ* (GCNIS) (Pryor *et al.*, 1983; Giwercman *et al.*, 1997; Petersen *et al.*, 1999; Olesen *et al.*, 2007), and prostate-specific antigen (PSA) (Ausmees *et al.*, 2014; Boeri *et al.*, 2021). The follow-up period was reported for all studies. PSA and GCNIS were the only outcomes evaluated concomitantly with the sperm analysis within cross-sectional design studies.

### Semen analysis methodology and discrepancies in reporting of results

Most studies reported on lab methodology for semen processing. Several retrospective studies included a large cohort over a period of time that used different cut-offs to define oligospermia (Glazer *et al.*, 2017b, 2019; del Giudice *et al.*, 2020; Chen *et al.*, 2022). Only seven studies reported a period of abstinence before providing semen sample between 2 and 7 days (Petersen *et al.*, 1999; Jensen *et al.*, 2009; Ausmees *et al.*, 2014; Glazer *et al.*, 2017b, 2019; Latif *et al.*, 2017, 2018). Groos *et al.* (2006) included all men with an abstinence period of <14 days.

### Pooling of data

Pooling of data with meta-analysis could be performed only for azoospermia and oligospermia categories associated with five outcomes: all-cause mortality, diabetes, CVD, all-site cancers, and testicular cancer. All other outcomes, e.g. GCNIS, PSA, or other comorbidities, as well as the value of other sperm parameters for prediction of long-term health were detailed in a narrative review as 2 × 2 tables could not be generated. A summary of results and detailed data extraction are found in Tables 2 and 3.

**Table 2.** Summary of findings from pooled analysis.

	Azoospermia vs oligospermia OR, 95% CI	Azoospermia vs normozoospermia OR, 95% CI	Oligospermia vs normozoospermia OR, 95% CI
All-cause mortality	OR 1.96, 1.29–2.96	OR 2.00, 1.23–3.25	OR 1.04, 0.52–2.09
Cardiovascular disease	OR 0.94, 0.74–1.20	OR 1.11, 0.71–1.75	OR 1.12, 0.80–1.55
Diabetes	OR 2.16, 1.55–3.01	OR 2.03, 0.67–6.13	OR 0.98, 0.37–2.59
All-site cancer	OR 2.16, 1.55–3.01	OR 2.18, 1.20–3.96	OR 1.05, 0.63–1.77
Testicular cancer	OR 1.53, 0.90–2.60	OR 1.80, 1.12–2.89	OR 1.25, 0.85–1.84

Calculated odds ratio (OR) with 95% CI for all analyzed outcomes comparing groups of azoospermic, oligospermic, and normozoospermic men.

**Table 3.** Pooled data used for meta-analysis of all outcomes.

	Azoospermia			Oligospermia			Normozoospermic/sterilized		
	Dead	Alive	Total	Dead	Alive	Total	Dead	Alive	Total
Jensen *et al.* (2009)	262	4163	4425				63	3493	3556
Glazer *et al.* (2019)	89	11 239	11328	407	75 769	76 176	99	18 263	18 362
del Giudice *et al.* (2020)	88	12 542	12 630	76	18 750	18 826	615	241 667	242 282
Keihani *et al.* (2020)	25	593	618	25	2190	2215	117	2909	3026
Eisenberg *et al.* (2014)				28	2607	2635	36	8682	8718
**Total**	464	28 537	29 001	536	99 316	99 852	959	164 055	165 014

	**CVD**	**No CVD**	**Total**	**CVD**	**No CVD**	**Total**	**CVD**	**No CVD**	**Total**
Latif *et al.* (2017)	91	375	466	146	445	691	703	2359	3262
Chen *et al.* (2022)	74	393	467	35	190	225	1101	8203	9304
**Total**	165	768	933	181	635	916	1804	10 562	12 566

	**Diabetes**	**No diabetes**	**Total**	**Diabetes**	**No diabetes**	**Total**	**Diabetes**	**No diabetes**	**Total**
Glazer *et al.* (2017b)	22	1253	1275	66	8472	8538	61	12 315	12 376
Latif *et al.* (2017)	21	445	466	16	675	691	127	3135	3262
**Total**	43	1698	1741	82	9147	9229	188	15 450	15 638

	**Cancer**	**No cancer**	**Total**	**Cancer**	**No cancer**	**Total**	**Cancer**	**No cancer**	**Total**
Jacobsen *et al.* (2000)	13	2662	2675	33	10 476	10 509	42	18 626	18 668
Eisenberg *et al.* (2013)	10	441	451	19	1768	1787			
Latif *et al.* (2017)	18	448	466	11	680	691	95	3167	3262
Keihani *et al.* (2020)	22	596	618	31	2184	2215	160	15 079	15 239
**Total**	63	4147	4210	75	13 340	13 415	316	38 640	38 956

	**TCa**	**No TCa**	**Total**	**TCa**	**No TCa**	**Total**	**TCa**	**No TCa**	**Total**
Jacobsen *et al.* (2000)	13	2662	2675	33	10 476	10 509	42	18 626	18 668
Latif *et al.* (2017)	9	457	466	9	682	691	45	3217	3262
**Total**	22	3119	3141	42	11 158	11 200	87	21 843	21 930

Total number of subjects extracted for every outcome and by semen concentration category.

TCa, testicular cancer; CVD, cardiovascular disease. Free cells indicate lack of data.

### The predictive value of semen concentration on long-term health in men

#### All-cause mortality

A total of five published studies (Groos *et al.*, 2006; Jensen *et al.*, 2009; Eisenberg *et al.*, 2014; Glazer *et al.*, 2019; del Giudice *et al.*, 2020) and one conference abstract (Keihani *et al.*, 2020) analyzed the correlation between suboptimal sperm parameters and the risk of death. Glazer *et al.* (2019) reported a higher risk of mortality in azoospermic men undergoing assisted reproduction (MAR) compared to non-MAR azoospermic (adjusted hazard ratio [aHR] 3.66, 95% CI: 2.18–6.16) and to MAR normozoospermic matches (aHR 2.40, 95% CI: 1.57–3.67). Similarly, del Giudice *et al.* (2020) observed a higher risk of mortality in azoospermic men (aHR 2.01, 95% CI: 1.60–2.53) compared to fertile controls. Oligospermia increased the risk of mortality in three of the studies: del Giudice *et al.* (2020), aHR 1.34, 95% CI: 1.01–1.79 in men over 35 compared to fertile controls; Jensen *et al.* (2009) observed the highest mortality ratio if concentration was less than 10 million/ml and Eisenberg *et al.* (2014) reported a higher risk of mortality in men with sperm concentration <15 million/ml, although this did not reach statistical significance. Two retrospective studies (Groos *et al.*, 2006; Keihani *et al.*, 2020) reported no increased risk of mortality in azoospermic and oligospermic men compared to normozoospermic or fertile controls.

**Figure 2.**
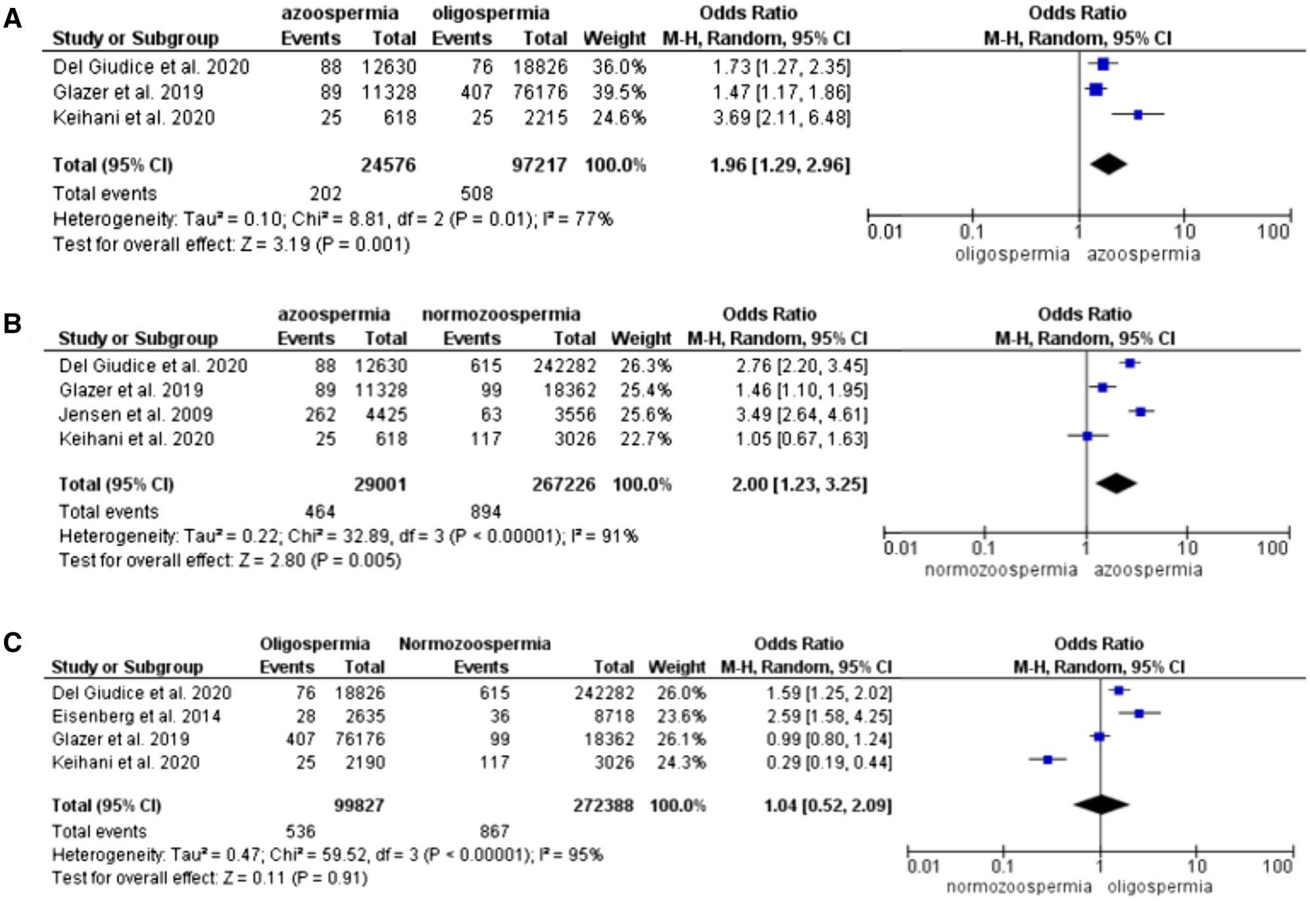
Forest plot (odds ratio, 95% CI, Mantel–Haenszel (M-H), random effect model) for risk of all-cause mortality between groups of men with (A) azoospermia and oligospermia, (B) azoospermia and normozoospermia, and (C) oligospermia and normozoospermia.

To analyze the overall effect of suboptimal sperm concentration on the risk of mortality, we were able to extract data in 2 × 2 form and perform meta-analysis from five papers. A total of 29 001 azoospermic men were compared to 165 014 normozoospermic/sterilized and 99 852 oligospermic men (Table 3). The maximum follow-up period among selected studies varied from 15 to 39 years. The risk of death was higher for azoospermic compared to normozoospermic/sterilized (OR 2.00, 95% CI: 1.23–3.25) and oligospermic men (OR 1.96, 95% CI: 1.29–2.96). There was no evidence of increased risk of death in the oligospermic group compared to normozoospermic/sterilized men (OR 1.04, 95% CI: 0.52–2.09) (Fig. 2).

#### Cardiovascular disease

**Figure 3.**
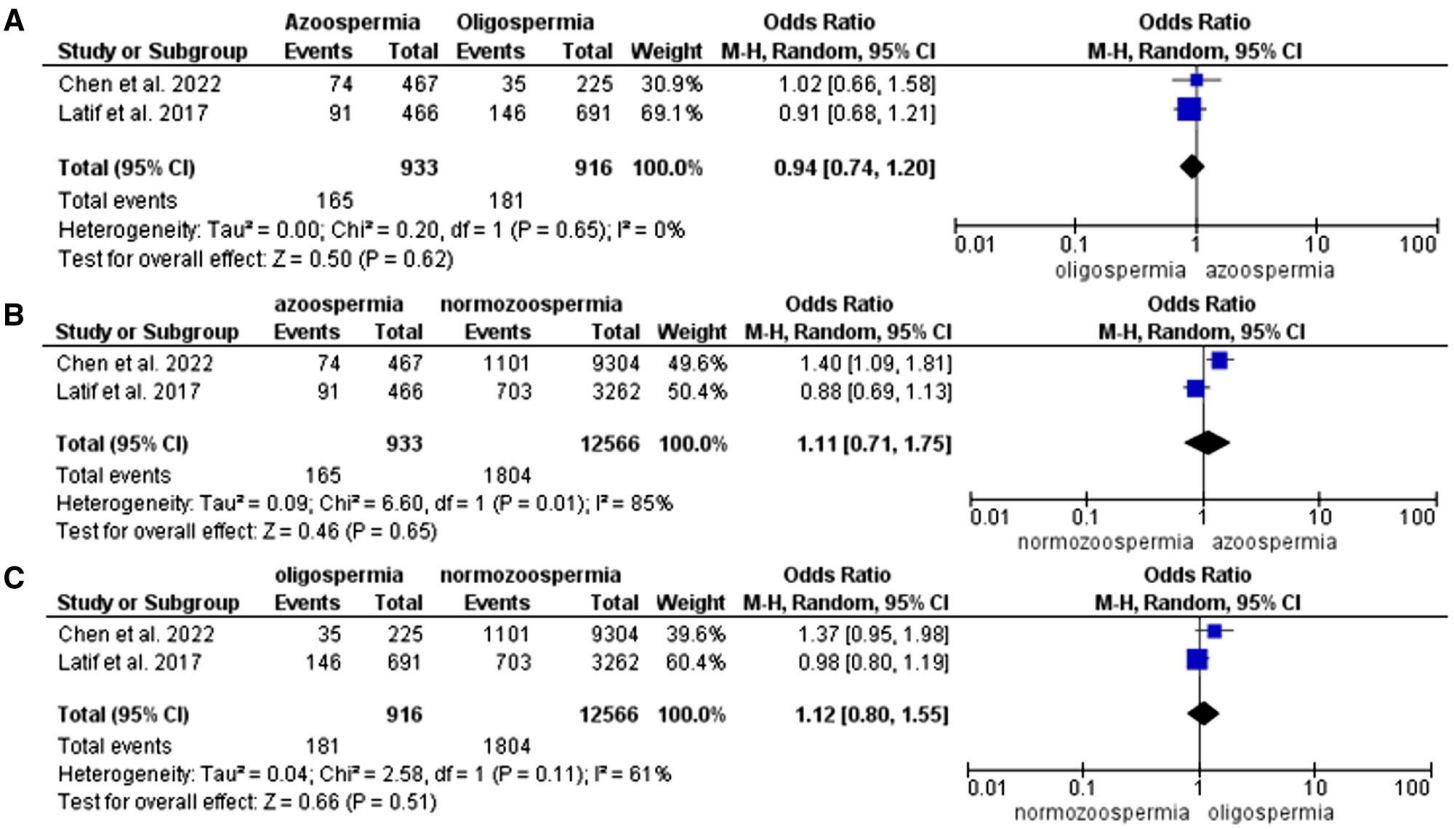
Forest plot (odds ratio, 95% CI, Mantel–Haenszel (M-H), random effect model) for risk of cardiovascular disease between groups of men with (A) azoospermia and oligospermia, (B) azoospermia and normozoospermia, and (C) oligospermia and normozoospermia.

Three studies were included for analysis of cardiovascular risk in relation to low sperm concentration. Latif *et al.* (2017) reported first-time hospitalization due to cardiovascular events. When adjusted for age and year of birth, aHR 1.1 (95% CI: 0.9–1.4) for azoospermia and aHR 1.4 (95% CI: 1.2–1.6) for oligospermia, compared to concentration >40 million/ml. Chen *et al.* (2022) found an increased risk in CVD: aHR for men with azoospermia 1.388 (95% CI: 1.221–1.584) and oligospermia 1.365 (95% CI: 1.191–1.562) compared to men without infertility. Eisenberg *et al.* (2016) did not observe a higher risk of cardiovascular events in azoospermic or oligospermic men compared to infertile men without impaired semen analysis. Only data from the first two studies could be cumulated and a total of 933 azoospermic, 916 oligospermic, and 12 566 normozoospermic/sterilized men were compared (Table 3). The results show no difference in CVD risk between azoospermic and oligospermic (OR 0.94, 95% CI: 0.74–1.20), azoospermic and normozoospermic (OR 1.11, 95% CI: 0.71–1.75), and oligospermic and normozoospermic groups (OR 1.12, 95% CI: 0.80–1.55) (Fig. 3).

#### Diabetes

**Figure 4.**
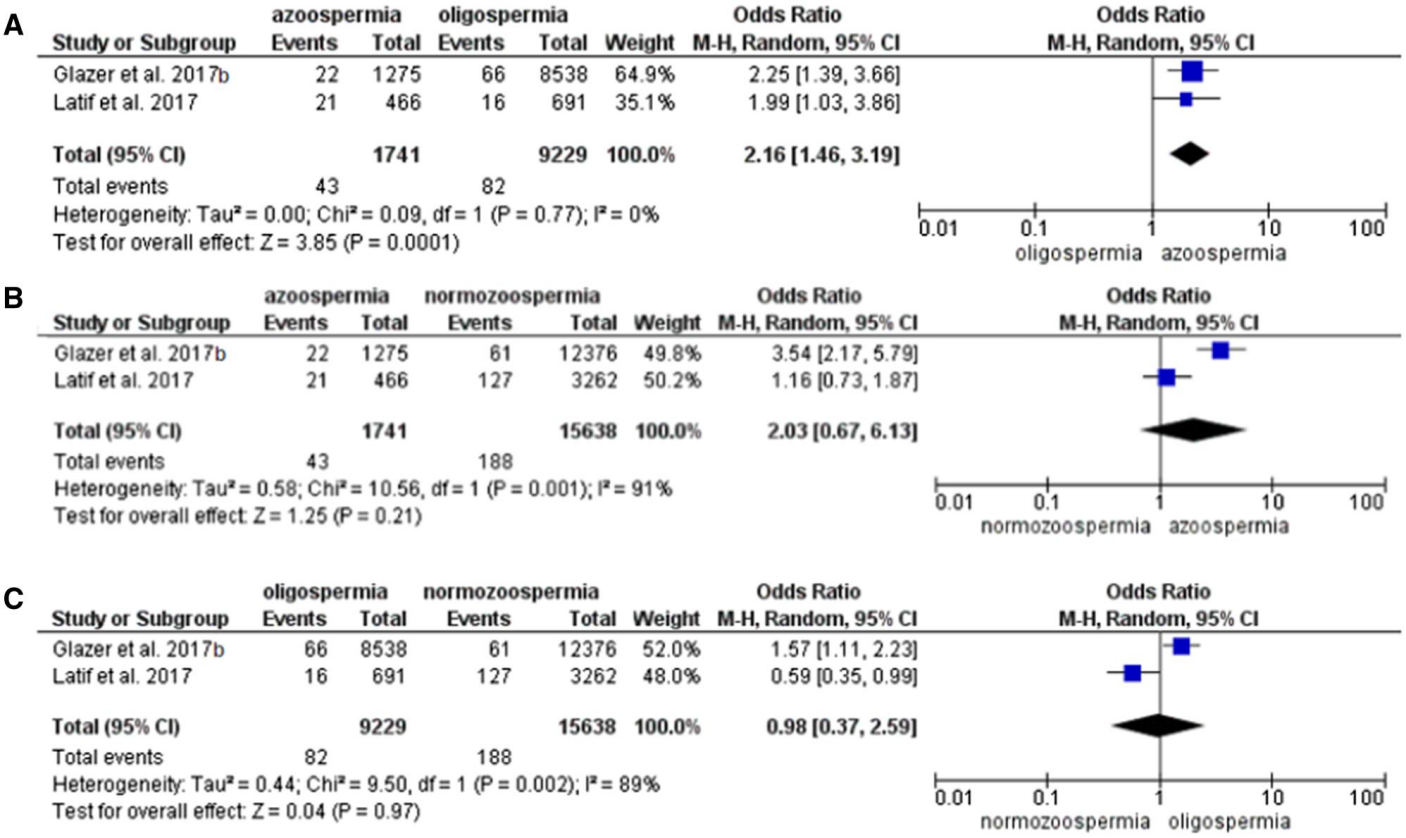
Forest plot (odds ratio, 95% CI, Mantel–Haenszel (M-H), random effect model) for risk of diabetes between groups of men with (A) azoospermia and oligospermia, (B) azoospermia and normozoospermia, and (C) oligospermia and normozoospermia.

Latif *et al.* (2017) observed 168 subjects with first-time hospitalizations due to diabetes. This excludes patients who were treated in outpatient clinics and only includes potentially more severe cases. The unadjusted HR compared to sperm concentration >40 million/ml was 1.9 (95% CI: 1.4–2.5) for azoospermia and 1.4 (95% CI: 1.1–1.8) for oligospermia. Similarly, Glazer *et al.* (2017b) found a higher risk of diabetes in both oligospermic (aHR 1.44, 95% CI: 1.01–2.06) and azoospermic men (aHR 2.10, 95% CI: 1.25–3.56) compared to the normozoospermic group. Eisenberg *et al.* (2016) found a higher risk of diabetes associated with oligospermia (aHR 1.33, 95% CI: 1.05–1.68). We could do a meta-analysis with the first two studies. A total of 1741 azoospermic men, 9229 oligospermic men and 15 638 normozoospermic men were compared (Table 3). The pooled analysis showed a higher risk of diabetes among azoospermic men compared to oligospermic (OR 2.16, 95% CI: 1.46–3.19), but no difference in risk between azoospermic and normozoospermic (OR 2.03, 95% CI: 0.67–6.13) or oligospermic and normozoospermic men (OR 0.98, 95% CI: 0.37–2.59) (Fig. 4).

#### All-site and testicular cancer

**Figure 5.**
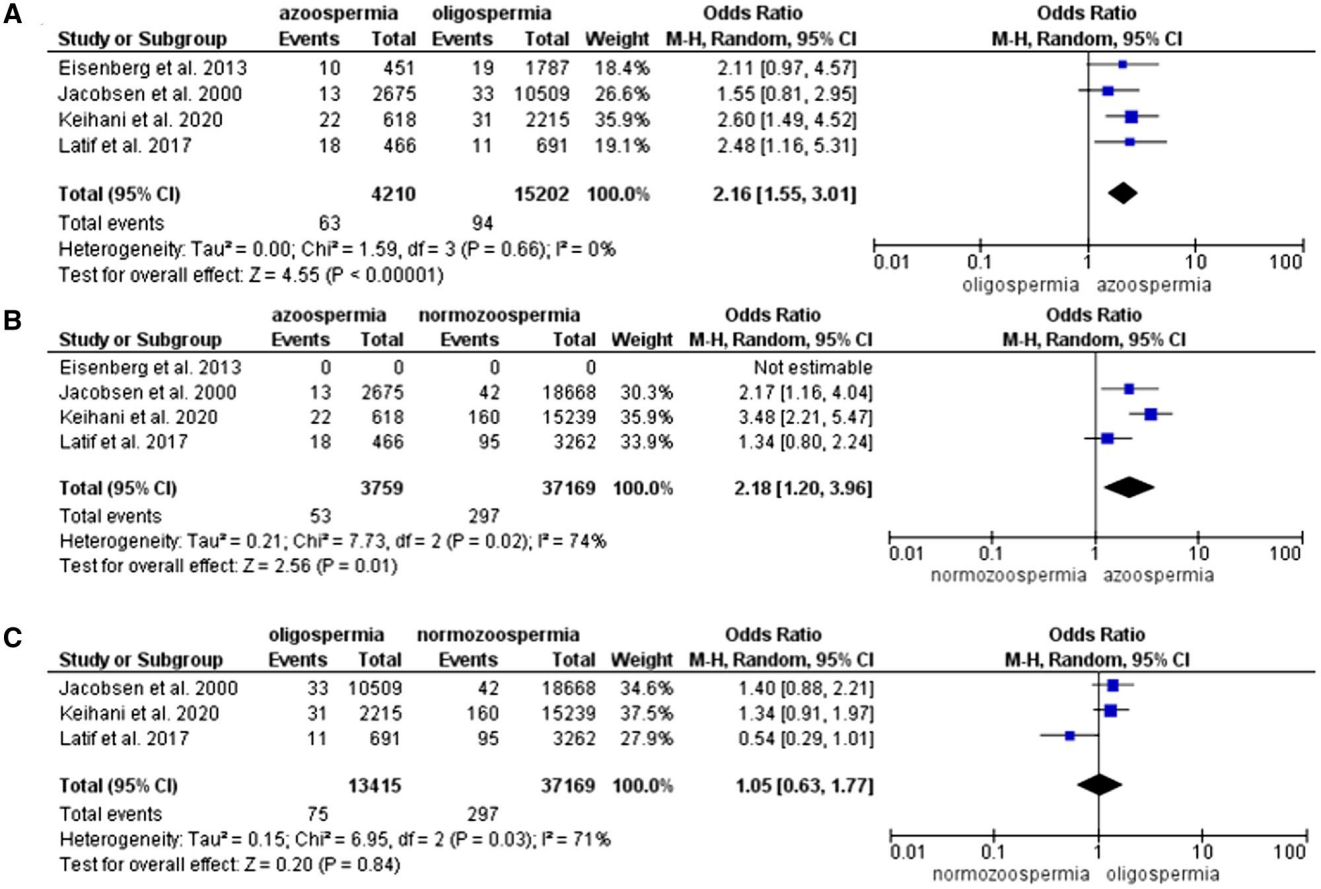
Forest plot (odds ratio, 95% CI, Mantel–Haenszel (M-H), random effect model) for risk of all-site cancer between groups of men with (A) azoospermia and oligospermia, (B) azoospermia and normozoospermia, and (C) oligospermia and normozoospermia.

Four studies were included for the analysis of cancer risk (Jacobsen *et al.*, 2000; Eisenberg *et al.*, 2013; Latif *et al.*, 2017; Keihani *et al.*, 2020). Three studies looked at all-cancer risk and two at the risk of testicular cancer in particular. Azoospermic men had a higher risk of all-site cancer compared to oligospermic (OR 2.16, 95% CI: 1.55–3.01) and normozoospermic (OR 2.18, 95% CI: 1.20–3.96) men, but there was no difference in risk between oligo and normozoospermic groups (OR 1.05, 95% CI: 0.63–1.77) (Fig. 5).

**Figure 6.**
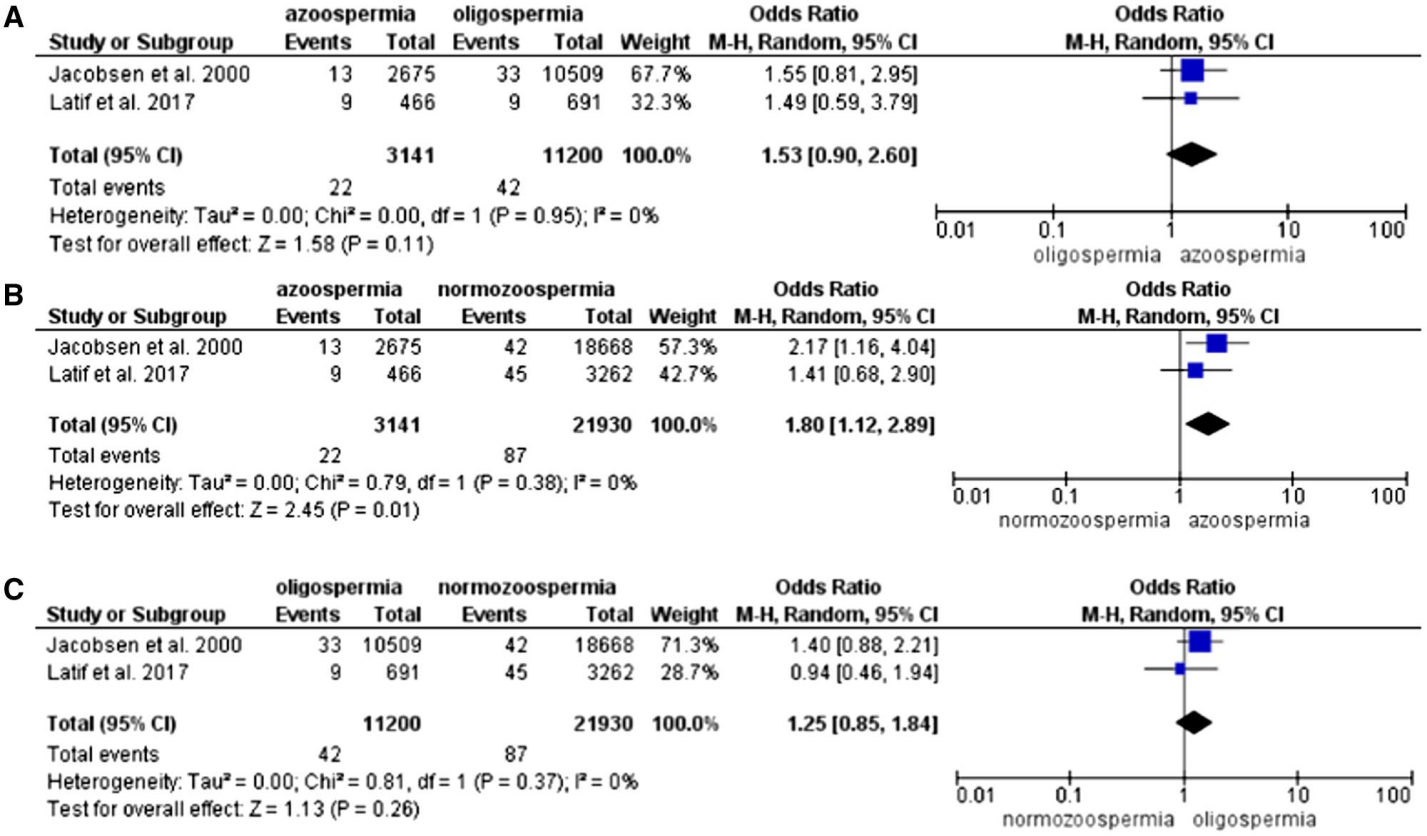
Forest plot (odds ratio, 95% CI, Mantel–Haenszel (M-H), random effect model) for risk of testicular cancer between groups of men with (A) azoospermia and oligospermia, (B) azoospermia and normozoospermia, and (C) oligospermia and normozoospermia.

Subgroup analysis showed that the risk of testicular cancer was higher in azoospermic men compared to the normozoospermic group only (OR 1.80, 95% CI: 1.12–2.89). There was no evidence that oligospermic men were at higher risk of testicular cancer (Fig. 6).

#### Germ cell neoplasia *in situ*

The term testicular carcinoma *in situ* (TCIS) has been recently substituted by the term GCNIS, therefore, we will refer to TCIS as being GCNIS. The highest risk of GCNIS has been reported in men with severe oligospermia with half of GCNIS at risk of developing invasive cancer in 5 years (Hoei-Hansen *et al.*, 2005). Pryor *et al.* (1983) found a 0.39% rate of GCNIS in 2043 infertile men where 7/8 men had a sperm concentration between 0 and 5 million/ml. Olesen *et al.* (2007) recruited men with poor semen analysis and reported a higher prevalence of GCNIS relative to an age-matched Danish population (2.2% vs 0.45%), which might be secondary to cohort selection. The median sperm concentration was almost the same in GCNIS-positive and negative groups. In another Danish cohort, (Giwercman *et al.*, 1997), no man with suboptimal semen had GCNIS on biopsy, but their cohort was very small. Petersen *et al.* (1999) looked at a population of men already undergoing orchidectomy for untreated unilateral testicular cancer. Subjects with GCNIS in the contralateral testicle had lower sperm concentration compared to the GCNIS-negative group. Because of the low prevalence of GCNIS in the general population, testicular biopsy cannot be implemented as a screening test and there is not enough data to suggest that severe oligospermia or azoospermia can be used as predictive tests for GCNIS to justify diagnostic testicular sampling.

#### Prostate-specific antigen and prostate cancer

PSA is used as a screening marker for prostate cancer but its value for decreasing prostate-specific mortality is small (Ilic *et al*., 2018). Four studies have investigated the association of semen analysis with the risk of prostate cancer or PSA value. Latif *et al.* (2017) observed 63 patients with prostate cancer. The risk of disease with azoospermia and oligospermia was higher in their cohort (HR 2.1, 95% CI: 1.5–2.9 and HR 1.5, 95% CI: 1.1–2.1, respectively). In the study from Ausmees *et al.* (2014), men with suboptimal semen parameters had higher PSA values than men with normal semen parameters. Boeri *et al.* (2021) analyzed the PSA values from 956 white European men attending for couple infertility evaluation and compared to 109 men with proven fertility. The median PSA was higher for the cohort of infertile men (0.7 vs 0.6 ng/ml, *P* = 0.02). Hanson *et al.* (2016) did not find an association between prostate cancer and azoospermia or oligospermia compared to fertile controls (HR 0.37, 95% CI: 0.04–3.26 and HR 0.53, 95% CI: 0.12–2.26, respectively). Based on the current data, semen analysis cannot be used to predict the risk of prostate cancer.

#### Hospitalizations and CCI

Two similar studies by Latif *et al.* (2018) looked retrospectively at all-cause first-time hospitalizations for 1423 men being investigated for couple infertility and showed that men with sperm concentration less than 15 million/ml had a higher risk of being hospitalized, but only when compared to a concentration >100 million/ml (HR 1.79, 95% CI: 1.53–2.11). Another 10-year prospective study (Boeri *et al.*, 2022) looked at the changes in health status based on the CCI of a highly selected cohort (899 non-Finnish, white European men non-inter-racial infertile couples). At 10 years follow-up, only 85 (9.5%) of infertile men had an increase in CCI and the majority (n = 44) had only one basepoint increase. Men with non-obstructive azoospermia showed a higher risk of having worsening CCI at their 5-year assessment, reaching a 30% risk of worsening CCI at 12-year follow-up. Subjects having a higher CCI also had a lower median and IQR sperm concentration (11.0, 0.0–28 million/ml vs 2.0, 0.0–14.0 million/ml). However, the intricacy of using CCI as a measurement tool is that it includes diseases that would be the result of accidents (hemiplegia, acquired immunodeficiency syndrome) or are more likely to happen later in life.

### The predictive value of other semen parameters on long-term health in men

Eight studies have assessed semen volume, motility, morphology, or vitality in relation to health outcomes (Petersen *et al.*, 1999; Jacobsen *et al.*, 2000; Jensen *et al.*, 2009; Ausmees *et al.*, 2014; Eisenberg *et al.*, 2014; Hanson *et al.*, 2016; Boeri *et al.*, 2021, 2022). Details of findings are shown in Table 1.

#### Cancer

Jacobsen *et al.* (2000) observed that men diagnosed with testicular cancer had a higher incidence of poor motility (standardized incidence ratio, SIR 2.5, 95% CI: 1.0–5.2, *P* < 0.05), morphology ≤25% (SIR 3.0, 95% CI: 0.8–7.6) and of having two (SIR 2.7, 95% CI: 1.1–5.5) or three (SIR 9.3, 95% CI: 1.0–33.4) abnormal sperm parameters. Similarly, Hanson *et al.* (2016) observed an increased risk of testicular cancer as motility, viability, and total motile count (TMC) declined (HR (Motile Trend) 1.3, 95% CI: 1.1–1.5; HR (Viability Trend) 1.3, 95% CI: 1.1–1.5; and HR (TMC Trend) 1.3, 95% CI: 1.1–1.6). Men in the lowest quartile for head morphology had an increased risk of testicular cancer (HR 4.2, 95% CI: 1.4–12.5). Men in the lowest quartiles of motility (0–49%), vitality (0–45%), and TMC (0–35 million) also had a higher risk of all-site cancer (HR 1.5, 95% CI: 1.1–2.0; HR 1.4, 95% CI: 1.0–1.9; and HR 1.6, 95% CI: 1.2–2.1, respectively). Petersen *et al.* (1999) did not observe a difference in median sperm volume (2.3 ml vs 2.9 ml, *P* = 0.09), median motility (57% vs 65%, *P* = 0.6), or median normal morphology (33% vs 36%, *P* = 0.62) between men with and without TCIS in contralateral testis at the time of orchidectomy.

#### Prostate-specific antigen

Ausmees *et al.* (2014) showed that the level of PSA for men with three normal semen variables (volume ≥1.5 ml, concentration ≥15x10^6^/ml, and A + B motility ≥40%) were significantly lower compared with subjects with altered parameters (1.51 ng/ml vs 2.47 ng/ml, *P* = 0.002). Men with PSA > 2.5 ng/ml had lower volume and sperm concentration but no difference in motility and men diagnosed with prostate cancer had only lower median total sperm count (205 million) and median concentration (81 million/ml) compared to controls with PSA <2.5 ng/ml (314 million and 105.5 million/ml, respectively). They also observed a negative correlation between semen volume and the level of PSA (*r* = 0.249, *P* = 0.005). Boeri *et al.* (2021) observed a lower motility only in men under the age of 40 years, with PSA >1 ng/ml compared to PSA ≤1 ng/ml (18% vs 29%, *P* < 0.001) with no difference in volume (3 ml vs 3 ml, *P* = 0.3) or incidence of normal morphology (2% vs 2%, *P* = 0.4).

#### Mortality

Jensen *et al.* (2009) observed in their cohort a trend for lower mortality risk, if semen volume (*P* trend 0.14), motility (*P* trend 0.04), and morphology (*P* trend 0.02) were improving. Eisenberg *et al.* (2014) analyzed two separate cohorts (California and Texas). In the California cohort, the risk of death was higher for sperm volume <1.5 ml (HR 2.17, 95% CI: 1.01–4.66) and in the presence of two abnormal sperm parameters (HR 2.64, 95% CI: 1.30–5.35) but there was no increased risk for motility <40%, TMC <9 million or normal morphology <14%. In the Texas population, the risk of death was higher only for TMC (HR 6.01, 95% CI: 1.77–20.41) and two abnormal sperm parameters (HR 12.22, 95% CI: 1.62–92.23). When populations were combined, the unadjusted HRs for death were higher for volume <1.5 ml (2.12, 95% CI: 1.19–3.77, *P* < 0.01), motility <40% (2.05, 95% CI: 1.24–3.37, *P* < 0.01), and the presence of two abnormal semen parameters (2.96, 95% CI: 1.67–5.25, *P* < 0.01). However, when results were adjusted for age and year of evaluation, the only statistically significant result was for men with two abnormal semen parameters (2.29, 95% CI: 1.12–4.65, *P* = 0.02).

#### Charlson Comorbidity Index

Boeri *et al.* (2021) did not observe lower median sperm volume (3 ml vs 3 ml), median motility (25% vs 17%, *P* = 0.3), or a lower percentage of patients with normal morphology (4% vs 4%, *P* = 0.5) in men who had an increase in CCI at follow-up.

No meta-analysis could be performed due to the heterogeneity of data.

## Discussion

### Main findings

Overall, our findings show azoospermic men to be at higher risk of all-cause mortality and cancer (all-site and testicular) when compared to normozoospermic and oligospermic men. There was no significant difference in risk of mortality and all-site cancer between men with oligospermia and normozoospermia. In particular, the risk of testicular cancer was found to be higher only in men diagnosed with azoospermia when compared to men with normal sperm concentration.

We found that the risk of being diagnosed with diabetes was higher in azoospermic men when compared to oligospermic men but both azoospermic and oligospermic groups had similar risk compared to normozoospermic men. This result is likely to be a spurious finding, mainly due to the small number of patients and the limitation to diabetes-related hospitalizations only in one of the studies.

The CVD risk analysis included a large variety of both cardiac and vascular pathologies. There was no data to create subgroup analysis by any specific diagnostic, limiting the validity of results and interpretation. The pooled results show no difference in risk of cardiovascular pathologies between any of the sperm concentration groups.

GCNIS and PSA are both pre-cancerous markers for testicular and prostate cancer, respectively. However, PSA can also be increased in other medical conditions. We looked at whether there is any evidence that poor sperm parameters might correlate to high PSA or the presence of GCNIS, and whether it could be further used to predict future risk of prostate or testicular cancer. However, the literature is insubstantial and no conclusions could be drawn.

The evaluation of other semen parameters in non-azoospermic men, in relation to the risk of developing comorbidities, was limited in all studies. Meta-analysis could not be performed due to large heterogeneity and variability of motility, morphology, or vitality in laboratory setting analysis.

It seems that semen volume, motility, and morphology are not reliable predictive factors and most studies have assessed them in isolation. However, the combination of multiple suboptimal semen parameters, as suggested in the study of Eisenberg *et al.* (2014), might have a better predictive value. This should be assessed in further studies.

### Strengths and limitations

The main strength of this review is that it takes into consideration solely the value of sperm parameters, rather than just the diagnosis of male infertility, in relation to health outcomes. Although the samples were analyzed in andrology laboratories, the practice is not devoid of flaws. Three quality control programs (Mallidis *et al.*, 2012; Filimberti *et al.*, 2013; Punjabi *et al.*, 2016) reported that some laboratories fail to adhere to recommended methods of semen analysis, and in 2012, the UK External Quality Assurance Scheme for Andrology (UK NEQAS) found significant variation in the analysis of the same sperm sample between laboratories (Pacey, 2013).

Moreover, the cut-offs of semen parameters were not consistent across all papers and the dose-related estimation of risk was not possible. Oligospermia was not consistently defined across studies. The cut-off would be set at either 15 or 20 million/ml and that was due to dynamic changes in the WHO manual across years. Another disadvantage is the use of a threshold level that does not permit assessment of the difference in risk between, e.g. 1 million/ml and 10 million/ml sperm concentration. Within the azoospermic group, there was also no differentiation between obstructive and non-obstructive azoospermic men, although the majority of papers excluded vasectomized patients. A few studies included sterilized men in the normozoospermic group, which is likely not to affect the overall results as the chances of having a fertile azoospermic/oligospermic patient going for vasectomy can be considered to be low. Eisenberg *et al.* (2013) separately analyzed a group of 101 patients where 91% were classified as non-obstructive.

Furthermore, we have made a rigorous risk of bias evaluation and we appreciate that this is subject to interpretation. In our view, none of the studies had a low risk of bias, mainly due to the retrospective nature of the studies and the constraints of data collection.

Due to these limitations, the results should be interpreted with caution.

### Comparison with other studies

We observed a higher risk of mortality in cases of azoospermia when compared to the other two groups, but not in oligospermic men. However, our results are not consistent with other studies. del Giudice *et al.* (2021a) showed a higher risk of death in both oligospermic men (HR 1.31, 95% CI: 1.11–1.54) as well as azoospermic men (HR 2.17, 95% CI: 1.55–3.05) compared to normozoospermic and no difference in risk of death in the azoospermic group compared to the oligospermic group (HR 1.39, 95% CI: 0.93–2.08). The difference was the result of an additional included study (Groos *et al.*, 2006, which we have not included as we could not use the crude data) and the difference in statistical method.

One study (Eisenberg *et al.*, 2016) of a large cohort of infertile and vasectomized men, suggested that infertile men might have an increased risk of diabetes (HR 1.30, 95% CI 1.10–1.53) and ischemic heart disease (HR 1.48, 95% CI 1.19–1.84). These hypotheses are supported by previous studies showing that men with a lower sperm concentration (<39 million/ml) are more likely to be diagnosed with metabolic syndrome (Ferlin *et al.*, 2021), subsequently placing them at higher risk of developing diabetes and cardiovascular events. Our pooled results only suggest a possible higher risk of diabetes in azoospermic vs oligospermic men, which is not a reliable conclusion based on the small sample of patients.

Men with azoospermia or severe oligospermia are usually evaluated with a testicular ultrasound scan but only a very small percentage are diagnosed with cancer (Mancini *et al.*, 2007). Previous systematic reviews only evaluated the risk of cancer associated with a diagnosis of infertility. del Giudice *et al.* (2020) estimated that infertile men are at increased risk of future testicular (RR 2.03, 95% CI: 1.66–2.47, *P* < 0.001, *I*^2^ = 87.12%) and prostate cancer (RR 1.67, 95% CI: 1.17–2.40, *P* = 0.005) compared to fertile controls. In contrast, a systematic review by Laukhtina *et al.* (2021), which included six extra studies, concluded that there is no evidence of correlation between infertile men and prostate cancer (OR 1.26, 95% CI: 0.63–2.54; *P* = 0.5, *I*^2^ = 100%). Interestingly, a meta-analysis looking only in the population of childless men (Mao *et al.*, 2016) found a lower risk of prostate cancer (OR 0.91, 95% CI 0.87–0.96, *P* < 0.001, *I*^2^ = 88.2%).

Based on semen concentration only, our results suggest that azoospermic men might be at a higher risk of all-site cancer compared to other groups, but the testicular cancer risk was increased only when compared against normozoospermic men.

### Implications for clinical practice and future research

Within the identified studies, there is significant heterogeneity, limiting the interpretation of pooled analysis. Apart from mortality and cancer risk in azoospermic men, there is limited data on the risk of developing other comorbidities in men with poor semen quality. At present, there is not enough evidence to advise men with poor semen analysis that they are at higher risk of long-term illness.

Further large studies are needed with linked semen analysis data, conducted as per WHO recommendations (World Health Organization, 2021). More detailed subgroup analyses by outcome are required. Control groups should be composed of men with normal semen analysis and, in parallel, comparison should be done with the general population. Semen analysis parameters should be used as a continuous variable rather than an arbitrary cut-off of 40 or 15. Although the 2021 WHO manual uses 95% CIs for data analysis, in a clinical setting, strict cut-offs are still being used to define a suboptimal semen sample, leaving little room for interpretation. Moreover, we predict that, when covering a large period of time, there is a considerable risk of inaccuracy in sperm parameters analysis given known intra- and inter-laboratory variability. We also know that parameters can vary in the same person with time. Where more than one sample is available from the same patient, this should be taken into consideration for analysis.

### Conclusion

While there are suggestions in the literature that suboptimal semen analysis or male infertility may be associated with long-term ill health, this systematic review shows that the studies available in the literature do not offer good enough quality evidence to support this notion. Hence, we believe that further large-scale, appropriately conducted studies, are needed before using semen parameters as predictors of mortality, diabetes, CVD, or cancer, in a clinical setting.

## Supplementary Material

hoae066_Supplementary_Data

## Data Availability

All extracted data from the original papers and the methodology of the review can be found in detail in the main paper and in Supplementary File S1. For any other information needed, the corresponding author can be contacted via email.
