# Supplementary material for: Lab-based semen parameters as predictors of long-term health in men—a systematic review
Source: Hum Reprod Open. 2024 Nov 8;2024(4):hoae066. doi: 10.1093/hropen/hoae066 (PMC11643900; doi:10.1093/hropen/hoae066)
Supplement: hoae066_Supplementary_Data [file hoae066_supplementary_data.docx]

**Supplementary File S1 -** Risk of bias and applicability assessment of twenty-one studies using QUADAS2 tool, with three questions for each one of the four domains.

1. **Patient selection**
2. How was the cohort sampled: consecutive or random sample of patients enrolled and is there a description of setting and population choice from database (from andrology/infertility center, single center study, definition of infertility)? -> is the cohort characteristic of the general population?
3. Was the reference group (fertile cohort) described?
4. Did the study have appropriate exclusion criteria (e.g.: vasectomy, history of cryptorchidism, history of testicular cancer, comorbidities) that would correlate with the outcome?
5. **Index test**
6. Was semen analysis available for all subjects?
7. If a cut-off was used, was it specified (e.g.: threshold for oligospermia, NOA≠OA, standard WHO manual)?
8. Mention of laboratory standards according to WHO manual
9. **Reference standard -> disease/outcome**
10. Defined outcome (by ICD)?
11. Competing risk
12. Adjusted (for comorbidities)
13. **Flow and timing**
14. Were all patients included in the analysis/followed-up?
15. Was there an acceptable follow-up period from semen analysis to outcome?
16. Censoring/missed from registry

NOA=Non-obstructive azoospermia; OA=Obstructive azoospermia; WHO=World Health Organization; ICD=International Classification of Diseases

Risk of bias assessment by study using QUADAS_2_ tool. *Legend: = high risk; = unclear risk; = low risk.*

| **Study** | **Year** | **Country** | **Type of study** | **Risk of bias** | **Applicability** |
| --- | --- | --- | --- | --- | --- |
| Pryor et al | 1983 | UK | Cross-sectional |  |  |
| Giwercman et al. | 1997 | Denmark | Cross-sectional |  |  |
| Petersen et al | 1999 | Denmark | Cross-sectional |  |  |
| Jacobsen et al. | 2000 | Denmark | Retrospective cohort |  |  |
| Groos et al. | 2006 | Germany | Retrospective cohort |  |  |
| Olesen et al. | 2007 | Denmark | Cross-sectional |  |  |
| Jensen et al. | 2009 | Denmark | Retrospective cohort |  |  |
| Eisenberg et al. | 2013 | USA | Retrospective cohort |  |  |
| Ausmees et al. | 2014 | Estonia | Cross-sectional |  |  |
| Eisenberg et al. | 2014 | USA | Retrospective cohort |  |  |
| Eisenberg et al. | 2016 | USA | Retrospective cohort |  |  |
| Hanson et al. | 2016 | USA | Retrospective cohort |  |  |
| Glazer et al. | 2017 | Denmark | Retrospective cohort |  |  |
| Latif et al. | 2017 | Denmark | Retrospective cohort |  |  |
| Latif et al. | 2018 | Denmark | Retrospective cohort |  |  |
| Glazer et al. | 22019 | Denmark | Retrospective cohort |  |  |
| Keihani, S et al. | 2020 | USA | Retrospective cohort (abstract) |  |  |
| Boeri et al. | 2021 | Italy | Cross-sectional |  |  |
| Del Giudice et al. | 2020 | USA | Retrospective cohort |  |  |
| Boeri et al. | 2022 | Italy | Prospective cohort |  |  |
| Chen et al. | 2022 | Taiwan | Retrospective cohort |  |  |
